# Improving Clinical Detection of Acute Lacunar Stroke: Analysis From the IST-3

**DOI:** 10.1161/STROKEAHA.119.028402

**Published:** 2020-04-09

**Authors:** Francesco Arba, Grant Mair, Stephen Phillips, Peter Sandercock, Joanna M. Wardlaw

**Affiliations:** 1 From the Stroke Unit, AOU Careggi, Florence, Italy (F.A.); 2 Division of Neuroimaging Sciences, Brain Research Imaging Centre, University of Edinburgh, United Kingdom (G.M., J.M.W.); 3 Brain Research Imaging Centre, SINAPSE Collaboration, United Kingdom (G.M., J.M.W.); 4 Centre for Clinical Brain Sciences, University of Edinburgh, Western General Hospital, United Kingdom (G.M., P.S., J.M.W.); 5 Division of Neurology, Department of Medicine, Dalhousie University and Nova Scotia Health Authority, Halifax, Nova Scotia, Canada (S.P.).

**Keywords:** brain, diagnosis, lacunar, sensitivity, specificity, stroke

## Abstract

We aim to identify factors associated with imaging-confirmed lacunar strokes and improve their rapid clinical identification early after symptom onset using data from the IST-3 (Third International Stroke Trial).

**Methods—:**

We selected patients likely to have lacunar infarcts as those presenting with: Oxfordshire Community Stroke Project lacunar syndrome; a random sample with National Institutes of Health Stroke Scale (NIHSS) score <7; and recent lacunar infarct identified on imaging by IST-3 central blinded expert panel. An independent reviewer rated brain scans of this sample and classified visible infarcts according to type, size, and location. We investigated factors associated with presence of lacunar infarct on a 24 to 48 hour follow-up scan using multivariable logistic regression and calculated sensitivity and specificity of Oxfordshire Community Stroke Project alone and in combination with NIHSS score <7.

**Results—:**

We included 568 patients (330 lacunar syndrome; 147 with NIHSS score <7; 91 with lacunar infarct on baseline imaging, numbers exclude overlaps between groups), mean (±SD) age, 73.2 (±13.6) years, 316 (56%) males, and median NIHSS score 5 (IQR, 4–8). On 24 to 48 hour scan, 138 (24%) patients had lacunar infarcts, 176 (31%) other infarct subtypes, 254 (45%) no visible infarct. Higher baseline systolic blood pressure (odds ratio, 1.01 [95% CI, 1.01–1.02]) and preexisting lacunes (odds ratio, 2.29 [95% CI, 1.47–3.57) were associated with recent lacunar infarcts. Sensitivity and specificity of lacunar syndrome was modest (58% and 45%, respectively), but adding NIHSS score <7 increased specificity (99%), positive and negative predictive values (97% and 87%, respectively).

**Conclusions—:**

In patients presenting within 6 hours of stroke onset, adding NIHSS score <7 to Oxfordshire Community Stroke Project lacunar syndrome classification may increase specificity for identifying lacunar stroke early after stroke onset. Our findings may help selection of patients for clinical trials of lacunar stroke and should be validated externally.

**Registration—:**

URL: http://www.controlled-trials.com/; Unique identifier: ISRCTN25765518.

## Abstract 2

Supplemental Digital Content is available in the text.

Stroke is the leading cause of disability in the world and a frequent cause of death, with ischemic stroke representing the majority of stroke subtypes. Lacunar stroke accounts for around 20% to 30% of ischemic strokes.^1^ Traditionally, lacunar stroke is thought to result from disease of a small perforating artery, with typical clinical syndromes,^2^ often leaving a small hole (ie, lacune) long term in the subcortical white matter. Given their distinct physiopathology,^3^ prompt identification of lacunar strokes may guide subsequent treatment and management.

The gold standard to identify acute lacunar strokes is magnetic resonance (MR) with diffusion-weighted imaging.^4^ However, MR is not widely available for acute stroke assessment, and computed tomography (CT), due to its accessibility, cost, and few contraindications is routinely used for acute stroke assessment. Unfortunately, small acute infarcts can be difficult to identify on CT and diagnosing patients with lacunar infarct in the acute stroke assessment may be challenging.

According to the Oxfordshire Community Stroke Project (OCSP) classification,^5^ the lacunar syndrome may be diagnosed with a combination of motor and sensory deficits, or with typical clinical syndromes (eg, dysarthria/clumsy hand). The OCSP classification has good interobserver reliability, may provide fast information about etiology and prognosis of acute stroke, and is easy to communicate among physicians.^6,7^ However, studies showed that while OCSP classification correctly identified nonlacunar strokes subtypes, sensitivity and specificity in identifying imaging-confirmed lacunar infarcts were inconsistent, particularly in the early hours after stroke onset.^8–10^

The IST-3 (Third International Stroke Trial)^11^ was a large multicentre trial of r-tPA (recombinant tissue-type plasminogen activator) in patients aged over 18 with any subtype of acute ischemic stroke. In a subgroup of the IST-3 trial, we tested the following: (1) factors associated with lacunar infarction on 24 to 48 hours follow-up CT scan; (2) OCSP classification in identifying subsequent lacunar infarction on 24 to 48 hour follow-up CT scan; and (3) combined OCSP and National Institutes of Health Stroke Scale (NIHSS) in identifying lacunar infarction on 24 to 48 hour follow-up CT scan.

## Methods

### Population and Procedures

The authors declare that all supporting data are available within the article (and the Data Supplement). IST-3 has a data access policy managed by contact with the investigators through University of Edinburgh website. We analyzed data from patients enrolled in the IST-3 trial. Briefly, IST-3 was a randomized, open-label trial of intravenous recombinant tissue-type plasminogen activator (0.9 mg/kg) versus control given within 6 hours of onset in patients with symptoms and signs of acute stroke in whom brain imaging had excluded hemorrhage or nonstroke lesions.^11^ Clinical examination included NIHSS and 8 easily recognizable clinical signs. The randomization system assigned the OCSP subtype according to a validated computer algorithm using such clinical signs, independently of the clinicians OCSP clinical syndrome diagnosis, to minimize bias in the assignment of the syndrome by knowledge of the imaging appearances by the treating clinician. All patients had prerandomization brain imaging with CT or MR, and a follow-up scan was performed 24 to 48 hours after randomization in all patients. All scans were assessed by a central expert panel blinded to clinical information.^12^

For the present study, we selected patients who were more likely to have a new lacunar stroke as having one or more of the following characteristics:

Patients with clinical diagnosis of lacunar stroke syndrome (LACS) at the time of study enrolment.
Patients with radiological diagnosis of recent lacunar infarct on the follow up (24–48 hours) scan according to the blinded expert panel ratings.
Patients with NIHSS score <7 as stroke severity considered less likely to have a large vessel occlusion and more likely to have lacunar stroke^13,14^ randomly selected from the 817 patients with NIHSS score <7 of whole IST-3 cohort. The selection was made manually with random patient anonymous number.

Each patient was present only once in each subgroup.

The IST-3 study was approved by local ethics committees and other regulatory bodies of all participating hospitals and institutions. All patients, or a relative if the patient lacked capacity, provided written consent.

### Imaging Analysis

In this analysis, we only used CT scans. All scans had already been scored for acute stroke and prestroke changes (eg, leukoaraiosis, atrophy, old stroke lesions) by the IST-3 expert panel, masked to clinical details. For the present analysis, a stroke neurologist, trained in CT readings (Dr Arba), independently rated all the prerandomization and follow-up scans blinded to all clinical information except the affected side and identified the supposed culprit ischemic lesion, when visible. A new lacunar infarct was defined as a round or ovoid-shaped hypodensity in the supratentorial subcortical white matter with maximum axial diameter ≤20 mm, not visible on or with increased hypodensity compared with the baseline scan. Supratentorial location of lacunar infarcts was categorized into thalamic, internal capsule, lentiform nucleus, and centrum semiovale. We excluded from the present analysis infratentorial new lacunar infarcts because of reduced CT quality in the posterior fossa. Recent basal ganglia infarcts with axial diameter >20 mm were defined as striatocapsular infarcts. Other infarct types were classified according to the IST-3 classification based on arterial territories and the amount of the territory affected.^12^ Two experienced neuroradiologists (Drs Wardlaw and Mair) cross-checked the neurologist’s scan readings and resolved uncertain cases.

### Statistical Analysis

We described the characteristics of the study population using descriptive statistics. To test differences between groups, we used χ^2^ Pearson test, Mann-Whitney *U* test or Kruskal-Wallis, ANOVA test as appropriate. We described differences among lacunar infarcts visible at the 24 hours scan according to lesion shape, location; and investigated factors associated with presence of lacunar infarct at the follow-up scan using multivariable logistic regression analysis adjusted for age, sex, NIHSS, and relevant variables with *P*<0.1 on univariable analysis. In the multivariable analysis, we considered a *P* value <0.05 as statistically significant.

We calculated sensitivity, specificity, positive and negative predictive values of OCSP lacunar classification in detecting visible lacunar stroke on the follow-up scan. We calculated a best scenario as the hypothetic case assuming that all patients with no lesion on the follow-up scan in fact had a lacunar infarct, and a worst scenario as the hypothetic case assuming that all patients with no lesion on the follow-up scan in fact had a nonlacunar infarct, and calculated sensitivity, specificity, positive and negative predictive values of OCSP lacunar classification (LACS) accordingly.

We therefore calculated a combined measure as follows: patients with OCSP consistent with both LACS and NIHSS score <7, scored=1, otherwise (nonlacunar OCSP or NIHSS score ≥7) scored=0. We also calculated the best scenario and the worst scenario sensitivity, specificity, positive predictive value and negative predictive values of the combined measure. Statistical analysis was carried out using SPSS for Windows (version 23.0; SPSS, Armonk, NY, IBM Corp).

## Results

From the whole IST-3 population (N=3035), we retrieved 332 patients with LACS, 92 patients with diagnosis of recent lacunar infarction on 24 to 48 hour scans according to the IST-3 expert radiological panel, and 147 (18%) randomly selected patients with NIHSS score <7 (N=817), giving a total of 571 individual patients with no overlap between the 3 groups. Three patients had no available CT scan and were therefore excluded from the analysis. This left 568 patients for the present study. A detailed flowchart of study population is shown in Figure. Overall, OCSP was as follows: total anterior circulation syndrome=35 (6%), PACS=161 (28%), LACS=330 (58%), POCS=42 (8%).

**Figure.**
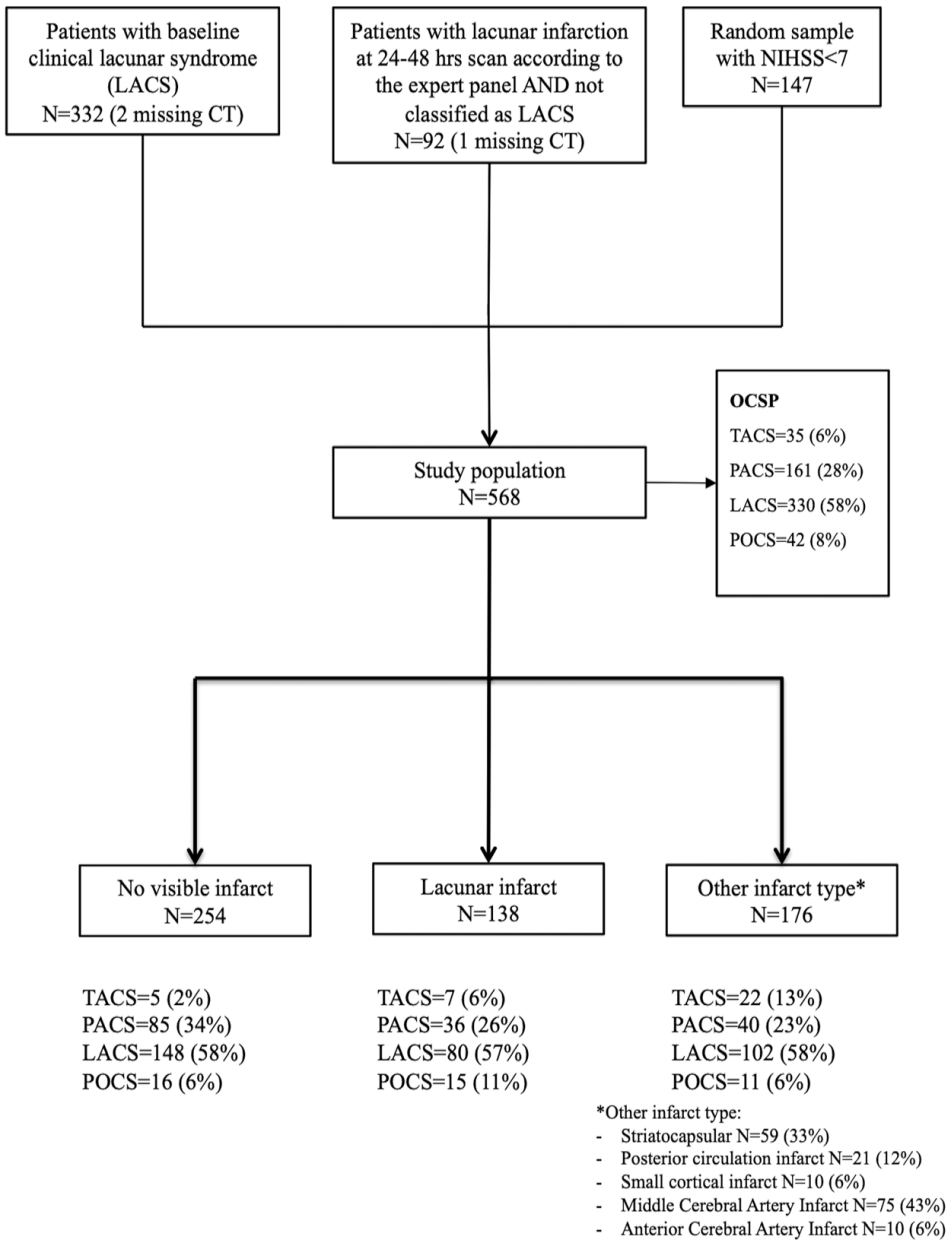
Diagram of the study population. CT indicates computed tomography; LACS, lacunar syndrome; NIHSS, National Institutes of Health Stroke Scale; OCSP, Oxfordshire Community Stroke Project; PACS, partial anterior circulation syndrome; POCS, posterior circulation syndrome; and TACS, total anterior circulation syndrome.

Characteristics of patients from each of the 3 groups are shown in Table 1. Patients with expert IST-3 radiological panel-diagnosed recent lacunar infarction had higher median NIHSS compared with LACS and random NIHSS score <7 sample (8 versus 6 versus 4; *P*<0.001), and more frequently presented with total anterior circulation syndrome syndrome compared with patients from the random NIHSS score <7 (26% versus 8%; *P*<0.001), whereas most patients with a clinical PACS syndrome were from the random NIHSS score <7 sample subgroup (76% versus 55%; *P*<0.001). There were no differences regarding prestroke radiological characteristics of leukoaraiosis or atrophy severity or presence of prior infarct. Among patients with LACS presentation (N=330, 58%), on the follow-up scan, 148 (45%) had no visible infarct, 80 (24%) had lacunar infarct, 102 (31%) had other infarct type. Lacunar infarct was more frequent in patients with higher NIHSS (6 for lacunar, 6 for other infarct type, 5 for no visible infarct; *P*<0.001), higher baseline glucose (mean, 132.9 mg/dL for lacunar; 125.6 mg/dL for other infarct type; 117.5 mg/dL for no visible infarct; *P*=0.021), higher systolic blood pressure (mean 165.1 mm Hg for lacunar; 151.1 mm Hg for other infarct type; 153.2 mm Hg for no visible infarct; *P*=0.016) and presence of preexisting lacunes (41% for lacunar; 25% and 26 % for other infarct type and no infarct, respectively, *P*=0.022; Data Supplement).

**Table 1.**
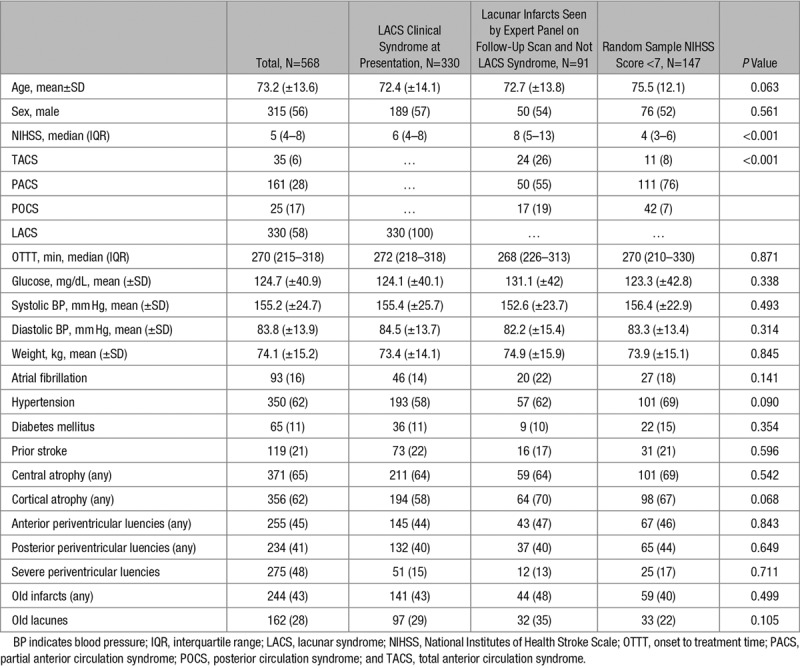
Clinical and Radiological Characteristics of the Study Population According to the Subgroup

In the whole study population, we identified 138 (24%) patients with recent lacunar infarct, 176 (31%) with other infarct type, and 254 (45%) with no visible new infarct (Table 2). Patients with any recent infarct had higher NIHSS scores (median 6 for other infarct type and lacunar infarcts, 5 for no infarct; *P*<0.001), whereas those with lacunar infarct had higher baseline systolic blood pressure (mean 160.8 mm Hg for lacunar infarct, 152.1 mm Hg for other infarct type, 154.3 for no visible infarct; *P*=0.016) and were randomized at later times (median 285 minutes for lacunar infarct, 264 minutes for other infarct type, 267 no infarct; *P*=0.046). Patients with recent lacunar infarcts had less severe leukoaraiosis grade (13% versus 21%, 9% for other infarct than lacunar; *P*=0.004) but more frequently evidence of preexisting lacunes (44% versus 21% for other infarct type and 26% for no infarct; *P*<0.001). After adjustment for confounders, we found that systolic blood pressure (odds ratio [OR], 1.01 [95% CI, 1.01–1.02]) and preexisting lacunes (OR, 2.29 [95% CI, 1.47–3.57]) were associated with lacunar infarct at follow-up. In the LACS patients subgroup, higher NIHSS (OR, 1.17 [95% CI, 1.03–1.32]), baseline glucose (OR, 1.01 [95% CI, 1.00–1.02]; *P*=0.018), systolic blood pressure (OR, 1.02 [95% CI, 1.01–1.03]), and preexisting lacunes (OR, 1.89 [95% CI, 1.06–3.38]) were associated with presence of lacunar infarct, whereas higher NIHSS (OR, 1.32 [95% CI, 1.15–1.50]) and preexisting lacunes (OR, 3.06 [95% CI, 1.50–6.24]) were associated with presence of lacunar infarct in non-LACS patients (Table 3).

**Table 2.**
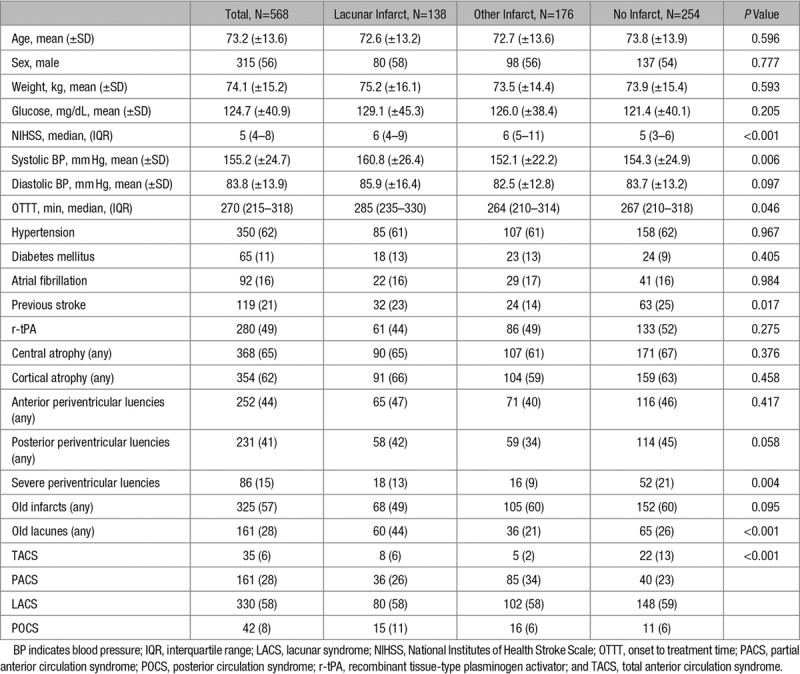
Characteristics of Study Population According to Radiological Findings of the Independent Reviewer at the Follow-Up Scan

**Table 3.**
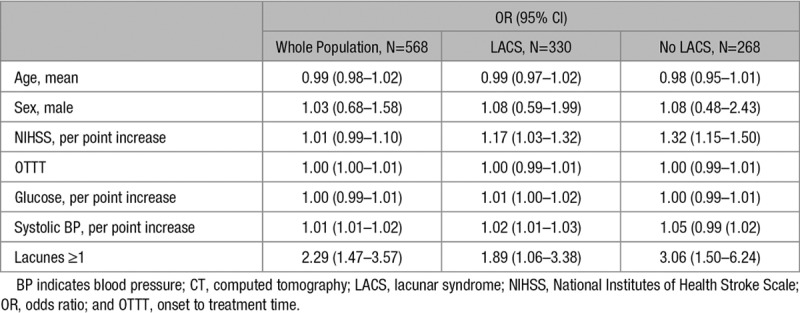
Multivariable Logistic Regression Showing Factors Associated With Presence of Lacunar Infarct on Follow-Up CT Scan

Among 138 patients with recent lacunar infarct, 86 (63%) had the infarct located in the basal ganglia (32 thalamus, 55 internal capsule/lentiform nuclei, 9 internal borderzone), whereas 52 (37%) in the centrum semiovale; 68 (49%) had round-shaped infarct and 70 (51%) ovoid; 97 (70%) had an axial diameter ≤15 mm and 41 (30%) an axial diameter from 15 to 20 mm (Data Supplement). Higher NIHSS was associated with larger lesion size; whereas patients with lesions ≤15 mm were older and more frequently had hypertension. Lacunar infarcts in the centrum semiovale had higher baseline systolic and diastolic blood pressure (Data Supplement). Further results in patients with subcortical versus lacunar infarcts, lacunar versus no infarct, follow-up scans of no-LACS patients and comparison of baseline factors associated with lacunar stroke in young and very old patients are shown in the Data Supplement.

LACS correctly identified 80/138 (58%) lacunar infarcts. Of the 59 (42%) remaining patients, 8 (6%) were classified as total anterior circulation syndrome, 36 as PACS (26%), 15 (11%) as POCS. Out of the 8 patients classified as total anterior circulation syndrome, 5 had old infarcts.

Table 4 shows sensitivity, specificity, positive predictive value and negative predictive value of LACS. Overall, accuracy of either LACS and NIHSS score <7 alone in diagnosing lacunar infarct was modest (sensitivity, 0.58; specificity, 0.45; sensitivity, 0.58; specificity, 0.31, respectively). If all patients with no visible infarct at the follow-up scan had a lacunar infarct (best scenario), positive predictive value of LACS moved from 0.27 to 0.71 and negative predictive value fall from 0.75 to 0.31; whereas if all patients with no visible infarct at the follow-up scan did not have a lacunar infarct (worst scenario), accuracy of LACS remained almost the same (positive predictive value 0.25 and negative predictive value 0.75). Similarly, sensitivity and positive predictive value of NIHSS score <7 increased with the best scenario and remained the same in the worst scenario.

**Table 4.**
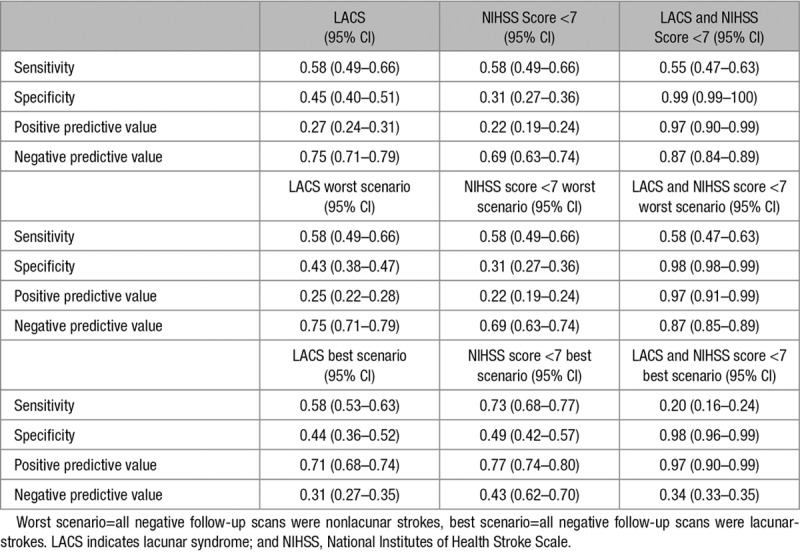
Sensitivity and Specificity for Lacunar Syndrome, NIHSS Score <7, and Lacunar Score in Detecting Lacunar Infarct

A total of 79 out of 138 patients fulfilled the combined LACS+NIHSS score <7 and did have a recent lacunar infarct. In the whole study population, the combined measure showed modest sensitivity (0.55) but excellent specificity (0.99), positive and negative predictive values (0.97, 0.98, respectively), which were confirmed in the hypothetical worst scenario. Sensitivity and negative predictive value of the combined LACS+NIHSS score <7 fell to 0.20 and 0.34 in the best scenario, but specificity and positive predictive value remained excellent (0.98 and 0.97, respectively).

## Discussion

In this population of likely lacunar stroke selected from a large multicenter clinical trial, we found that one-fourth of patients had a relevant lacunar infarct as seen by follow-up CT scan, whereas almost a half of patients did not have imaging evidence of infarction. Higher baseline blood pressure and preexisting lacunar infarcts were associated with presence of new lacunar infarct on the follow-up CT scan. Although OCSP had poor sensitivity and specificity for detection of lacunar infarct, a combined clinical measure using LACS and NIHSS remarkably improved specificity.

Identification of lacunar stroke with plain CT is challenging and may potentially lead to false negative findings. Consistent with previous studies that showed negative CT scan in 35% and 50% of patients with lacunar stroke,^10,15,16^ we found a negative CT scan in 45% of our population. In line with previous studies,^17,18^ the characteristics of younger and older patients with lacunar infarcts were slightly different to those of the general population. We identified clinical factors associated with presence of a new lacunar lesion on follow-up CT including higher systolic blood pressure in the whole study population, NIHSS score <7, and higher blood glucose in the LACS subgroup. Similar to our results, previous studies identified systolic blood pressure as variably associated with subsequent lacunar infarction,^19,20^ and diabetes mellitus but not baseline blood glucose was also associated with lacunar infarction.^21^ Among radiological factors, preexisting lacunes were associated with a new lacunar infarction both in LACS and non-LACS populations. This is in keeping with another study that found leukoaraiosis more common in patients with lacunar strokes and silent brain infarction,^22^ suggesting a common origin and risk factors of lacunar lesions throughout the brain.

Similar to previous studies, OCSP alone missed around a half of lacunar strokes investigated with CT,^15,23^ with low sensitivity and specificity, underlining the limits of CT in diagnosing small cerebral infarcts but also the modest accuracy of OCSP.^22,24^ On the other hand, we found that low stroke severity intended as NIHSS score <7 was not able to reliably identify lacunar strokes. However, we showed that combining clinical features assessed with OCSP and stroke severity may improve early identification of subsequent lacunar strokes. While the sensitivity of our combined measure was still modest, the specificity remarkably increased compared with LACS and NIHSS alone. In other words, with our combined clinical assessment, we were able to exclude strokes other than lacunar. Considering the limited capacity of CT scan in diagnosis of lacunar stroke, we hypothesized a worst scenario (ie, all negative follow-up scans were not lacunar strokes) and a best scenario (ie, all negative follow-up scans were lacunar strokes), and specificity of the combined measure remained the approximately same in both scenarios. This finding highlighted the importance of a careful clinical examination and assessment of acute stroke, particularly for suspected lacunar stroke subtype, since also angio-CT and perfusion CT do not provide additional information in diagnosis of lacunar infarction during the acute assessment.^25,26^ MR could identify a greater proportion of stroke compared with CT scan; however, a significant proportion of false negative findings remains.^26–29^ Particularly for future pragmatic trials targeted on lacunar stroke, a practical screening tool allowing reliable identification of lacunar stroke with limited use of MR imaging would be useful.

Our study has limits. The use of CT rather than MR for diagnosis of ischemic lesion may underestimate the real burden of new ischemic lesions and we probably missed useful information, although MR is not perfect in this respect^29^ either, and not all the subcortical small cerebral infarcts consistent with lacunar infarcts may evolve into an established lacune.^30,31^ However, the specificity and positive predictive value of the combined measure was excellent in the best and worse scenario, lending support to a careful clinical examination of patients with suspected lacunar stroke. As a subgroup analysis of a randomized controlled trial, we cannot exclude an effect of r-tPA, on the fate of the ischemic lesion; however, we did not find relevant differences in r-tPA use among patients with different infarct type. The NIHSS cutoff we adopted was arbitrary, although based on previously reported probability to exclude a large vessel occlusion^13^ and data from a large study on lacunar strokes.^14^ However, different NIHSS thresholds may be explored with statistical methods to increase sensitivity of the combined measure. Again, our results arise from a retrospective study and are therefore hypothesis generating, and need external validation in other cohorts, possibly using MR as imaging technique to identify a greater number of lacunar infarcts.

In conclusion, we showed that in patients presenting within 6 hours of symptom onset with clinical features suggestive of a lacunar stroke syndrome, systolic blood pressure and the presence of preexisting lacunes on the first CT scan were associated with an increased likelihood of detecting an acute lacunar infarct on a CT scan repeated 24 to 48 hours later. An indicator combining the clinical features of a lacunar syndrome (according to the OCSP classification) with stroke severity (defined as NIHSS score <7), increased the specificity of clinical lacunar stroke diagnosis. If validated, our results may find practical application in the diagnosis of hyperacute lacunar stroke and help select patients for clinical trials targeted at lacunar stroke.

## Acknowledgments

The IST-3 (Third International Stroke Trial) collaborative group thanks all patients who participated in the study. We gratefully acknowledge the members of the trial steering committee, image reading panel, national coordinators (Appendices I and II in the Data Supplement).

## Sources of Funding

IST-3 (Third International Stroke Trial) was funded from a large number of sources (see Appendix II in the Data Supplement) but chiefly the UK Medical Research Council (MRC G0400069 and EME 09-800-15) and the UK Stroke Association. The work was supported by the UK Dementia Research Institute which receives its funding from DRI Ltd, funded by the UK Medical Research Council, Alzheimer’s Society and Alzheimer’s Research UK, the British Heart Foundation Centre for Research Excellence Award III (RE/18/5/34216), the European Union Horizon 2020, PHC-03-15, project No 666881, ‘SVDs@Target’, the Fondation Leducq Transatlantic Network of Excellence for the Study of Perivascular Spaces in Small Vessel Disease, ref no. 16 CVD 05, and the Row Fogo Centre for Research into Ageing and the Brain, Ref No: AD.ROW4.35. BRO-D.FID3668413. Dr Sandercock received grants from UK Medical Research Council and from UK Stroke Association. Boeringher and Ingheleim donated the drug and the placebo for the pilot phase of the IST-3 study.

## Disclosures

None.

## Supplementary Material


